# Light-Up RNA Aptamers and Their Cognate Fluorogens: From Their Development to Their Applications

**DOI:** 10.3390/ijms19010044

**Published:** 2017-12-23

**Authors:** Farah Bouhedda, Alexis Autour, Michael Ryckelynck

**Affiliations:** Architecture et Réactivité de l’ARN, CNRS, Université de Strasbourg, UPR 9002, F-67000 Strasbourg, France; f.bouhedda@ibmc-cnrs.unistra.fr (F.B.), a.autour@ibmc-cnrs.unistra.fr (A.A.)

**Keywords:** light-up aptamer, fluorogen, fluorogenic dye, fluorescence, RNA, in vitro evolution, gene expression monitoring, live-cell imaging, biosensing

## Abstract

An RNA-based fluorogenic module consists of a light-up RNA aptamer able to specifically interact with a fluorogen to form a fluorescent complex. Over the past decade, significant efforts have been devoted to the development of such modules, which now cover the whole visible spectrum, as well as to their engineering to serve in a wide range of applications. In this review, we summarize the different strategies used to develop each partner (the fluorogen and the light-up RNA aptamer) prior to giving an overview of their applications that range from live-cell RNA imaging to the set-up of high-throughput drug screening pipelines. We then conclude with a critical discussion on the current limitations of these modules and how combining in vitro selection with screening approaches may help develop even better molecules.

## 1. Introduction

Quoting the famous idiom: “seeing is believing”, which makes imaging and spectroscopic technologies very popular in life sciences to detect and track molecules. Among the different visualization strategies, fluorescence is the most attractive and widely used, mainly because it is safe, sensitive and it offers the possibility of analyzing multiple colors at the same time, with a long shelf life of the fluorophores when properly stored. Among the different fluorescent probes, those acquiring their fluorescence only in permissive conditions (e.g., presence of a target molecule) and hereafter defined as being fluorogenic are the most useful. These probes exhibit only a weak fluorescence in non-permissive conditions, making washing steps dispensable, and permitting the monitoring of dynamic processes in time course experiments. A plethora of such fluorogenic molecules has been developed for applications as diverse as ions and small molecules sensing, pH monitoring or environment viscosity assessment [1,2]. Moreover, significant efforts have been devoted to the development of fluorogenic tools dedicated to RNA and protein, two biological polymers particularly relevant to cellular activity. Historically, these molecules were first detected using fluorescently labeled and extracellularly supplied specific probes such as antibodies and oligonucleotides [3]. Recent developments allow these strategies to reach single-molecule sensitivity, but the requirement of fixing cells represents a major drawback that could be overcome using genetically encoded fluorescent reporters.

The discovery of the naturally fluorescing Green Fluorescent Protein (GFP) from the jellyfish *Aequorea victoria* [4] had a profound impact on protein imaging field and paved the way for major breakthroughs. Indeed, this first genetically encoded probe post-translationally acquires its fluorescence through an autocatalytic cyclization involving three amino acids (Serine, Tyrosine, and Glycine) independently of any cell factor [5,6], making it usable in any cell type. Moreover, simple point mutations can shift the excitation/emission spectra of the protein toward the blue [7] or the red [8] regions of the visible spectrum. These great properties, shared with many other fluorescent proteins (FPs), make them highly versatile (see [9] for a comprehensive review) and very attractive for biotechnological applications. However, the strict requirement of molecular oxygen for the maturation of their fluorophore also limits FPs application in some circumstances and led to the development of alternative labeling strategies in which a fluorescent dye labels the protein of interest via a peptide (e.g., tetracystein peptide labeled by the Fluorescein Arsenical Helix binder FlAsH [10]) or a whole domain (e.g., SNAP-tag labeled by dye conjugated to a benzylguanine group [11]) appended to the target protein [12].

In contrast to proteins, no naturally fluorescent RNA has been discovered yet, making the development of RNA-based genetically encoded fluorescent reporters less straightforward than their protein counterparts. A first live-cell compatible strategy pioneered by Bertrand et al. [13] consists of inserting tandem repeats of elements recognized by an RNA-binding protein (RBP) into the RNA to image. Then, co-expressing this construct with an RBP-GFP fusion protein allows the direct labeling of the target RNA with the GFP. Moreover, the use of a split form of the GFP allows converting the otherwise always fluorescent GFP into a fluorogenic system in which fluorescence is expected only upon RBP-GFP/target RNA interaction [14]. Whereas this approach proved to be efficient for live-cell monitoring of large messenger RNAs [15], it may be more challenging for smaller RNAs (e.g., regulatory RNAs). Indeed, the insertion of a large number (20–30) of RBP binding sites into a small target RNA as well as its later decoration by RBP-GFPs leads to a significant increase of RNA size that could interfere with its biological function, making a size reduction of the labels a high priority. As was the case for protein labels, significant size reduction can be obtained by exchanging the bulky GFP for a smaller fluorescent synthetic dye. Constructs in which tandem repeats of RNA aptamers (i.e., small nucleic acid folds able to specifically recognize a target molecule) specific to a dye can be inserted into the target RNA, and the labeling can be obtained by placing cells in culture medium containing a membrane permeable dye. Aptamers binding specifically to fluorescent dyes such as sulforhodamine, fluorescein [16] or modified cyanines [17] can be used in arrays [17,18]. However, since the dye emits fluorescence even in its free unbound form, such approach may suffer from significant background fluorescence, limiting its application spectrum. Nevertheless, this limitation can be overcome by exchanging the fluorescent dye for a fluorogenic one. In this view, a seminal work by Tsien’s group showed that, not only an RNA aptamer can specifically interact with a target molecule, but this interaction can also strongly increase the fluorescence of compounds such as Malachite green, making such aptamer/dye couple fluorogenic [19]. This discovery was all the more astonishing that the Malachite green-binding aptamer was not originally selected to function as a light-up aptamer but rather to mediate site-specific inactivation of target RNAs [20]. Since then, a variety of fluorogenic dyes and their cognate RNAs have been developed [21] (Table 1). This short review will be primarily focused on the main design strategies of each partner (the dye and the RNA) prior to giving a rapid overview of their application scope ranging from in vivo live-cell RNA imaging to in vitro small molecule biosensing. For a more general view of the current technologies available to image RNA, the reader is redirected to other reviews [22,23,24,25].

## 2. Development of RNA-Based Fluorogenic Modules

RNA-based fluorogenic modules are made of two components: a fluorogenic dye (later called fluorogen) and a specific light-up RNA aptamer. In an ideal case, such module should feature five essential properties. (i) The module should be as bright as possible to ensure sensitive detection, ideally down to single molecule resolution. Therefore, the dye should have an elevated absorption coefficient (ε) and its interaction with the RNA should place it in a conformation and an environment maximizing its quantum yield (Φ^bound^) that should be as close as possible to 1. (ii) In its free form, the fluorogen should display the lowest possible quantum yield (Φ^free^) to minimize background fluorescence. Consequently, a good fluorogenic module is expected to have the highest possible fluorescence enhancement (calculated as the ratio of Φ^bound^ over Φ^free^). (iii) The RNA–dye interaction should be highly specific and bio-orthogonal (i.e., cell compounds or reagents of the assay should not interfere with the interaction). (iv) Moreover, the interaction should occur with a high affinity (dissociation constant *K_D_* in the nM range or less) to make using low concentration of the dye possible and to keep fluorescence background as low as possible, which allows getting high contrast [37]. (v) Finally, the module should be as photostable as possible to allow prolonged data acquisition. Since both components of the module affect all these parameters (with the exception of the second one that is only affected by the dye), the proper development of an RNA-based fluorogenic module is not a trivial task and both the dye and the RNA should be developed while trying to stay as close as possible to the ideal scenario depicted above.

### 2.1. Fluorogenic Dye Engineering

The fluorogen plays a key role in the functionality of a fluorogenic module by contributing to both its brightness and its photostability. Moreover, for live-cell applications, additional parameters such as membrane permeability and the lack of toxicity of the dye should also be considered. The fluorogenicity of a dye can be obtained in various ways encompassing but not limited to charge (or proton) transfer, conformational change, isomerization or even dye aggregation [38]. Several of these principles having been used for the development of RNA-based fluorogenic modules.

**Figure 1:**
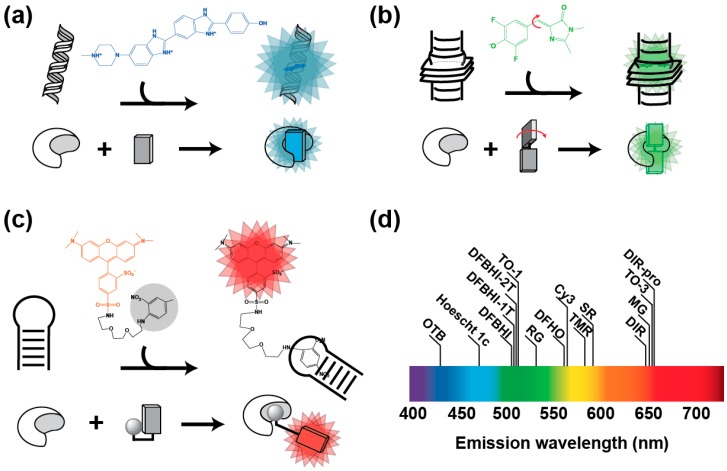
Main conception strategies of fluorogens. (**a**) Environment sensitive fluorogens. Molecules such as Hoescht 33258 (formula in blue) emit blue fluorescence upon association with DNA minor groove. Modifying the fluorogen with bulky groups allows aborting this non-specific DNA binding capacity, while preserving the fluorogenic capacity that can be now specifically activated by the cognate aptamer [35]. (**b**) Molecular rotor fluorogens. DFHBI (formula in green) [29] eliminates excitation energy by molecular movements (red arrows). However, upon association to the cognate aptamer, movements are restricted and fluorogen energy is eliminated by fluorescence emission. (**c**) Quenched fluorogens. Sulforhodamine B (formula in red) fluorescence is quenched by a conjugated dinitroaniline (formula in black and shaded in gray) [32]. However, the fluorescence is restored upon the specific recognition of the quencher (or the fluorophore moiety in other systems) by an aptamer. In every example, RNA aptamer is represented by the croissant-shaped object and the fluorogen by the rectangles. (**d**) Distribution of fluorogen emission wavelength along the visible spectrum.

Many compounds are known to become highly fluorescent upon interaction with nucleic acids [39]. Among them, ethidium bromide [40] and Hoechst 33258 [41] are environment sensitive dyes characterized by a poor fluorescence in aqueous solution. However, upon binding to a DNA molecule (respectively by intercalation between base pairs or binding in the DNA minor groove at AT-rich region), the dye is placed into a non-polar environment that strongly increases its fluorescence (Figure 1a). The non-specific DNA binding capacity of these dyes can lead to significant unwanted background fluorescence when used in cell-based assay. However, this property can be suppressed by decorating the dye with additional chemical functions. For instance, substituting Hoechst 33258 with bulky groups allowed designing derivatives no longer capable of non-specific DNA binding, but preserving their fluorogenic capacity and becoming fluorescent only upon specific interaction with DNA and RNA aptamers [28,42] (Table 1).

Fluorogenicity can also result from intramolecular movements allowing for non-radiative relaxation of the dye upon excitation (Figure 1b). Such compounds, hereafter called molecular rotors, are poorly fluorescent in their unbound form in a fluid environment. However, fluorescence can be restored by restricting intramolecular movements either by strongly increasing medium viscosity, or upon specific interaction with a nucleic acid. Triphenylmethane dyes (e.g., Malachite Green (MG) and Patent Blue, Table 1) were the first class of compounds for which specific RNA light-up aptamers were identified [19,20]. Among them, MG shows an impressive ~2400-fold fluorescence enhancement when bound to its cognate RNA aptamer [19]. Nevertheless, MG light irradiation is known to elicit the generation of free radical leading to subsequent RNA cleavage [20], making this approach potentially toxic for living cells [34,43,44]. Moreover, MG was also found to be able to generate significant background fluorescence within mammalian cell [45] and bacteria [46], further limiting the use of this system. Unsymmetrical fluorogenic cyanines constitute a second attractive class of fluorogenic molecular rotors composed of two different heterocycles connected by a methine bridge subjected to twisting in fluid solution [47,48]. Thiazole Orange (TO) is a good representative of these dyes, but it is also characterized by a significant non-specific DNA binding capacity [49,50]. However, as with Hoechst 33258, modifying the dye makes it possible to strongly attenuate this adverse effect. Indeed, using a large dimethylindole heterocycle and substituting the quinolone ring with a propylsulfonate group led to Dimethyl Indole Red (DIR), a TO derivative displaying a strongly reduced non-specific interaction with nucleic acids [36]. Alternatively, adding an acetate group on the benzothiazole moiety led to TO-1, a TO derivative also displaying a reduced affinity for DNA [33]. Moreover, a specific RNA aptamer was developed for each TO derivative (Table 1). In addition to their elevated absorption coefficient, cyanines are attractive molecules from a spectral point of view since their excitation/emission spectra can be easily modulated by changing the length of the methine bridge. Indeed, simply lengthening this bridge by two carbons converted the green emitting TO-1 into a red-emitting TO-3 [33]. Despite these promising properties, one can also foresee that current cyanine-derived dyes may suffer a potential limitation regarding cell-permeability. Indeed, the charged (e.g., acetate and sulfonate [27]) and bulky polar groups (e.g., PEG-biotin [33]) used to prevent non-specific binding, may also affect the capacity of the dyes to freely cross the cell membrane. Nevertheless, transiently caging these groups (e.g., by protecting carboxylic group as acetoxymethyl (AM) esters) may improve their membrane permeability. A last class of molecular rotors is made of GFP-mimicking dyes, a set of synthetic fluorogens that mimic the fluorophore of the GFP formed upon the cyclization of the Ser-Tyr-Gly tripeptide. Many derivatives of these fluorogens have been synthesized and used to develop a variety of sensors (see [51] for a recent review). Among them, the 3,5-difluoro-4-hydroxybenzylidene imidazolinone (DFHBI, Table 1) was developed by Jaffrey’s group in 2011 together with a specific light-up aptamer [29]. Among other interesting features, DFHBI displays a ~1000 fluorescence enhancement in the presence of specific binding aptamers, it is non-toxic and has good cell permeability. Further substituting the imidazolinone cycle with a trifluoroethyl or a pentafluorophenyl group allowed obtaining DFHBI-1T [30] and DFHBI-PFP [45], two dyes with improved brightness and spectral properties. Whereas these GFP-mimicking dyes emit in the green region of the spectrum, the recent development of the DsRed-mimicking dye 3,5-difluoro-4-hydroxybenzylidene imidazolinone-2-oxime (DFHO, [34]) and others [52] also makes possible imaging nucleic acids in the orange-red region of the spectrum.

Finally, fluorogenic dyes can be obtained by appending a quenching group to a fluorescent organic dye (Figure 1c). In its free off-state, the fluorescence of the dye is quenched by a mechanism of photoinduced electron transfer (PET, [53,54]), Förster resonance energy transfer (FRET, [35]) or contact-mediated quenching [32,55]. However, the presence of an aptamer specifically recognizing either the dye [16,55] or the quencher [32,35,53] moiety allows for retrieving the fluorescence of the dye and the emission of a bright fluorescence (Table 1).

All together, these different strategies allowed designing a variety of fluorogens with fluorescence emission spanning most of the visible spectrum (Figure 1d) and brightness sometimes exceeding that of the broadly used GFP (Table 1).

### 2.2. Isolation of Fluorogenic RNA Aptamers

Once a fluorogen has been designed, the molecule can be used as target for the isolation and the optimization of a specific light-up RNA aptamer using two conceptually different approaches: (i) a selection strictly speaking during which an RNA pool is challenged to interact with the dye and only the best binders are recovered; or (ii) a screening approach where the light-up capacity of each molecule is analyzed and only the most fluorogenic ones are recovered.

#### 2.2.1. Aptamers Selection Based on Binding Capacity

Historically, RNA (or DNA) aptamers are isolated using the Systematic Evolution of Ligands by EXponential enrichment (SELEX) approach [56,57], a set of technologies particularly well suited for the identification of nucleic acid specifically recognizing virtually any type of target, ranging from ions to more complex proteins [58]. Conceptually, SELEX works by performing iterative rounds of selection to exponentially enrich an RNA (or DNA) library in molecules able to bind a target molecule. Moreover, SELEX possesses the attractive capacity of handling large sequence diversity (10^12^ to 10^15^ sequences) in a single experiment.

**Figure 2:**
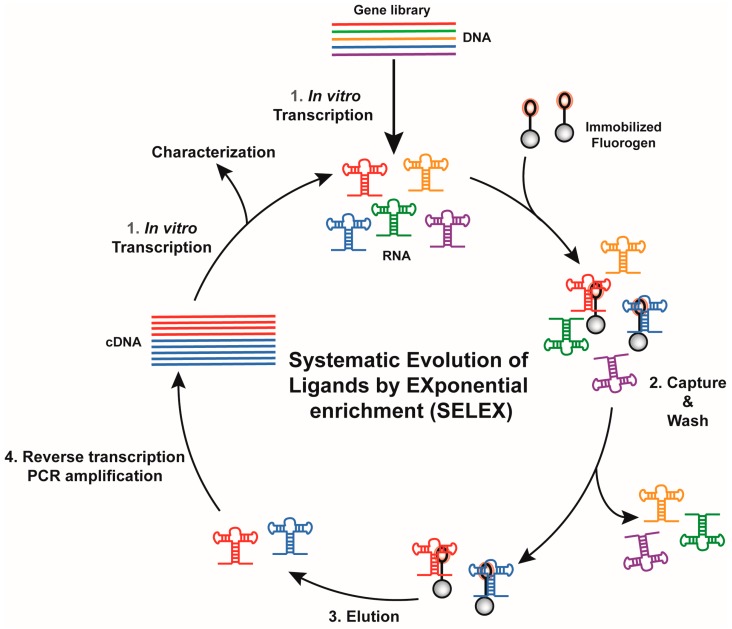
General overview of an in vitro selection using Systematic Evolution of Ligands by EXponential enrichment (SELEX). Each round proceeds in four main steps. A gene library is in vitro transcribed (Step 1) and mixed with beads displaying the fluorogen. Poor binders are eliminated by wash of variable stringency (selection pressure, Step 2) prior to recovering binding RNAs during the elution step (Step 3). Finally, RNAs are converted into cDNAs (Step 4), later used to prime a new round of selection.

Depending on target size and physicochemical properties, several variations of SELEX have been introduced. However, all the new light-up RNA aptamers isolated so far were obtained through the same selection scheme (Figure 2). The success of such selection relies mainly on the proper design of the starting library as well as the partition mode used during the selection process. The starting DNA gene library is usually obtained by inserting a randomized region of ~50 nucleotides or more between two constants regions. Moreover, early work on the selection of aptamers targeting small molecules showed that interrupting the randomized region with a short constant hairpin may increase the success rate of a selection process [59] and, interestingly, the use of such discontinuous randomized libraries allowed isolating some of the best fluorogenic aptamers described so far [27,29,31,36]. The selection process is then initiated by transcribing the DNA gene library into an RNA pool prior the selection step where target-binding RNAs are partitioned away from the non-binding ones. To do so, the RNA pool is usually incubated with beads (e.g., streptavidin-conjugated agarose or magnetic beads) displaying the fluorogenic dye attached via a PEG linker. After several washing steps, the retained RNAs are eluted and reverse transcribed into cDNAs. These cDNAs can be later PCR-amplified and used to prime a new round of selection. Gradually increasing the wash stringency (e.g., increase the number and/or the duration of the wash) during the selection process allows favoring the selection of aptamers with the highest affinity for the dye while the poorer binders are counter selected. Moreover, the choice of the elution mode may also significantly affect the properties of the selected molecules. For instance, immobilized RNAs can be eluted using an excess of free dye. Such a general selection scheme led to the isolation of several fluorogenic RNAs such as the Malachite Green-binding aptamer [20], the DFHBI-binding aptamer Spinach [29], the DFHO-binding aptamer Corn [34] and the Dimethylindole Red-binding aptamer [27,36]. However, even though competition-based elution may favor the isolation of aptamers having great specificity for the dye moiety, they also suffer the major drawback that variants with low dissociation constant, so very high affinity, might likely be counter selected and lost. This may partly explain why these aptamers have a moderate affinity (*K_D_* ~0.1–1 µM) for their cognate dyes. This limitation could be overcome by changing the elution mode. For instance, the insertion of a disulfide bond within the linker between the dye and the bead, allows for the elution of dye/RNA complexes by adding dithiothreitol [32]. Alternatively, retained RNAs can be recovered by eluting them under denaturing conditions (e.g., heating, addition of urea, formamide, NaOH, etc.) as used for the selection of Hoechst-binding aptamer [28] and TO-1-binding Mango RNA aptamer [33]. Combined with drastic washing conditions (ionic strength reduction and addition of free competitive dye in the washing buffer), denaturing elution allowed the isolation of Mango RNA, a fluorogenic aptamer forming a fluorescent complex with the thiazole orange derivative TO-1-biotin characterized by a *K_D_* in the nM range, the highest affinity described so far for a fluorogenic module (Table 1). Nevertheless, one should note that RNAs isolated by non-competitive elution might also display affinity not only for the dye moiety but also accessory elements. This is well exemplified by Mango RNA that recognizes the TO-1-biotin not only via its thiazole orange moiety, but also displays some affinity for the PEG linker and the terminal biotin [60].

Upon isolation, fluorogenic aptamers can be further optimized by truncation and rational design. For instance, rationally inserting a few mutations in the Spinach aptamer resulted in Spinach2, a DFHBI-binding aptamer with improved folding properties [61]. However, some beneficial mutations may be difficult to predict and improving these aptamers may require individually testing many mutants. Moreover, an intrinsic limitation of SELEX is linked to the way selections are performed. Indeed, in this format, molecules are selected for their capacity to bind a fluorogen rather than for their capacity to trigger its fluorescence. Therefore, screening approaches, in which molecules are directly analyzed for their fluorogenic capacity, represent an attractive complement to SELEX.

#### 2.2.2. Aptamers Isolation Based on Their Fluorogenic Capacity

**Figure 3:**
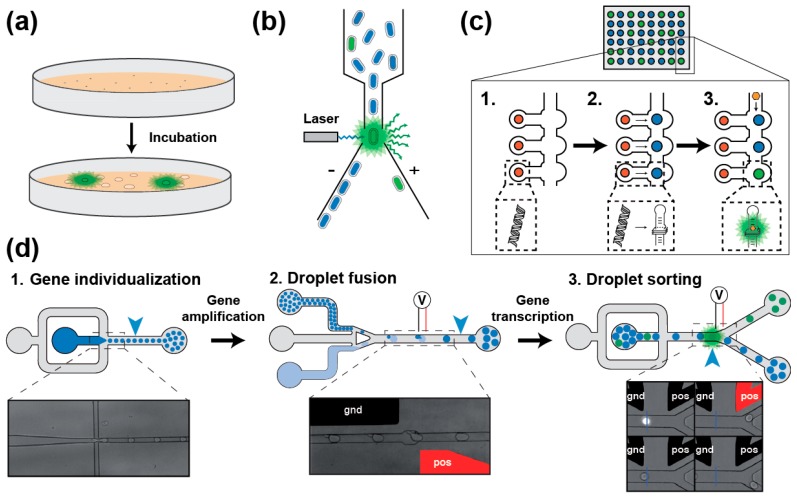
Main screening strategies for isolating light-up RNA aptamers. (**a**) Colony screening [31,62]. SELEX-enriched gene library is cloned and expressed in bacteria plated on a solid medium supplemented with the fluorogen. Upon incubation, colonies expressing light-up aptamers are identified by fluorescence emission (green shadow) when illuminated with fluorogen excitation wavelength. (**b**) FACS-based bacteria screening [31]. SELEX-enriched gene library is cloned and expressed in bacteria incubated in the presence of the fluorogen. Bacteria fluorescence is then analyzed on a FACS using a laser exciting the fluorogen. Fluorescence emitting bacteria (green shadow) are then deflected and sorted from the rest of the population. (**c**) Miniaturized in vitro screening using large integration scale microfluidic devices [64]. DNA coding for each variant is first spotted (red dots, Step 1) onto a surface prior to assembling the microfluidic chip. Then, each DNA cluster is transcribed in RNA later captured on a second spot of the chip (blue spot, Step 2). Finally, flowing the fluorogen into the microfluidic channels (Step 3) allows the detection of light-up aptamers (green spot). Quantifying the fluorescence emitted by each construct at various concentrations of fluorogen allows the determination of parameters such as the brightness and the dissociation constant (*K_D_*). (**d**) Droplet-based microfluidic in vitro screening workflow [65]. A gene library is diluted into a PCR mixture (in dark blue) prior to individualizing the molecules into picoliter-sized water-in-oil droplets carried by an oil phase (in gray, Step 1). Upon off-chip PCR amplification, small and amplified DNA-containing droplets (dark blue) are synchronized and fused with larger droplets containing an in vitro transcription mixture supplemented with the fluorogen (light blue, Step 2). Upon an incubation step allowing for in vitro transcription to take place, the fluorescence of each droplet is analyzed and those droplets containing a light-up aptamer (in green) are sorted from the rest of the population (in blue). Both fusion and sorting events are triggered by the application of an electric field to built-in positive (pos, shown in red) and ground (gnd, shown in black) electrodes.

Screening a light-up aptamer gene library requires isolating each gene prior to its expression into RNA followed by the assessment of its light-up capacity. In a first scheme, the library can be inserted into an expression plasmid later used to transform bacterial cells that will express light-up aptamers [31,62]. Bacteria can then be screened in two ways. First, they can be plated onto a solid medium supplemented with the fluorogen and allowed to grow until forming colonies [62]. The plates are then observed under fluorogen exciting light and the most fluorescent clones are recovered (Figure 3a). Even though it is simple and direct, such approach suffers from a limited throughput (a few thousand clones per day) and exploring large libraries can rapidly become time consuming, tedious and expensive. Alternatively, upon gene expression the cells can be incubated with the fluorogen prior to being screened using a Fluorescence-Activated Cell Sorter (FACS) (Figure 3b) [31], which substantially increases the analytical throughput (up to several million genes analyzed per day). However, even though it is more accurate, these screening approaches explore a much smaller fraction of the sequence space than SELEX does. Therefore, preferred methods combine library pre-enrichment by SELEX followed by ultrahigh-throughput screening using FACS. Such a tandem procedure was already used to obtain different light-up aptamers such as Broccoli [31], a new DFHBI-binding molecules [63] and Corn [34]. These in vivo approaches offer the great advantage of directly assaying the performance of the aptamers in the cellular context, and in doing so to select those RNAs with optimal in vivo performances. However, they also suffer two major drawbacks: (i) the absolute need of using a cell-permeable fluorogen (a property dispensable for in vitro applications); and (ii) the applicable selection pressures are limited to those compatible with living cells (e.g., presence of potassium, physiological pH and temperature). Moreover, in the case of FACS, since the analysis is performed at single cell level, the screening may also face cell-to-cell expression variability and requires the co-expression of an internal fluorescent standard (e.g., GFP and mCherry).

Most of the cell-based screening limitations can be overcome by switching to an in vitro expression approach. Performing such screening in microtiter plate can rapidly become very expensive, making the miniaturization of the process using microfluidics highly desirable [66]. A first level of miniaturization can be achieved by using a large-scale integration microfluidic device made of 640 independent one-nanoliter chambers, each containing several copies of the DNA coding for the variant to assay (Figure 3c) [64]. Then, genes are in vitro transcribed into RNA, which are later individually assayed for fluorogenic capacity. Moreover, the possibility of varying ligand concentration during the experiment allows the extraction of thermodynamic parameters of each variant in an automated way. However, the low throughput of the method (640 chambers per run) mainly restricts its use for refinement purposes. Further substantial throughput increase and cost reduction can be achieved by transposing the screening to droplet-based microfluidics [65,67] (Figure 3d). In this format, DNA molecules of a library are diluted into a PCR mixture and individualized at very high throughputs (several thousands of droplets generated per second) in small two-picoliter water-in-oil droplets. Upon thermocycling, each small droplet is fused to a larger 18-picoliter droplet containing an in vitro transcription mixture supplemented with the fluorogen. Finally, upon a last incubation, the fluorescence of each droplet is profiled and the most fluorescent ones (so those containing the best light-up aptamers) are sorted. Using this approach, we recently isolated iSpinach, a variant of the Spinach light-up aptamer optimized for in vitro applications (far less salt-sensitive, brighter and more thermo-stable) [65]. The isolation of this variant was made possible by the great control over the reaction conditions and the possibility of applying strong selection pressures difficult to apply in cell-based screening (e.g., warming and complete replacement of potassium ions by sodium). Therefore, droplet-based microfluidics screening is a viable and efficient way of isolating optimized light-up aptamers for in vitro application. Finally, further automation of the process could be obtained by integrating a Next Generation Sequencing analysis at the end of the process.

### 2.3. Features of the Best Characterized RNA-Based Fluorogenic Modules

Among the variety of RNA-based fluorogenic modules developed so far (Table 1), those involving MG, fluorescent protein mimicking dyes (e.g., DFHBI and DFHO) and TO-1 are the best characterized, especially from a structural point of view. Interestingly, even though each of these RNAs adopts a distinct folding, they all possess a fluorogen-binding pocket comprising an extended planar platform made of at least one base quadruple onto which the fluorogen is accommodated in a near planar conformation competent for efficient fluorescence emission (see [68] for a recent critical review on the topic). Whereas the MG-binding aptamer platform is made of mixed base quadruple [69], the platform of all the other light-up aptamers is made from a stack of two or more G-quartets stabilized by potassium ions [60,70,71,72,73], indicating that such platform might be a consensus solution to fluorogens binding. However, beside this consensus platform, the rest of the fluorogen-binding pocket as well as the recognition strategy vary from one aptamer to the other. Indeed, for instance, MG-binding pocket acquires its structure only upon dye binding. On the other hand, in Spinach and its derivatives, the DFHBI intercalates between the platform and a base triple whilst additional contacts are established with lateral bases [70,71,72]. This pocket is formed prior to dye binding and was found to accommodate other dyes [74] limiting the possibility of using these aptamers concomitantly to other ones in multiplex experiments. Moreover, the pocket does not strongly constrain the dye that is free to rapidly isomerize upon illumination followed by a rapid exchange with a non-isomerized fluorogen to restore the fluorescent module [75]. DFHBI-based fluorogenic modules are therefore poorly photostable under continuous-wave illumination and are better imaged using a pulsed mode illumination scheme. This behavior challenges the accurate monitoring of some biological phenomena (e.g., RNA movement in the cell). Moreover, even though no crystal structure has been solved yet for Broccoli (another DFHBI-binding aptamer), the strong sequence homologies as well as photophysical behavior shared with Spinach suggest that both aptamers are very likely to possess an identical fluorogen-binding pocket [76]. Recently, the structure of Corn RNA in complex with DFHO has been resolved [73] and revealed that, surprisingly, the fluorogen-binding pocket does not lie into a single RNA molecule but instead is formed by the interface of two interacting protomers of identical sequence, each made of four base quadruples and six adenine residues asymmetrically oriented. This preformed binding pocket can specifically accommodate a single DFHO and not only triggers its fluorescence but it also constitutes a highly protective environment preserving the dye from photobleaching over a long period of time [34]. Consequently, even though Corn/DFHO module is dimmer than the other systems, its strong photostability makes it well suited for the prolonged imaging of RNAs in live cells. Finally, the crystal structure of RNA Mango in complex with TO-1 helped explain in part the origin of the elevated affinity in the complex. Indeed, like the other aptamers, Mango possesses a planar platform made of G-quartets and interacts not only with the cyanine moiety but also with the PEG linker and the biotin group of the fluorogen [60]. Interestingly, both heterocycles of the cynanine are not coplanar but instead they make a 45 °C angle, suggesting that brighter mutants of the aptamer that would accommodate the cyanine more coplanarly could, in principle, be isolated by functional screening (a work currently in progress in our lab).

Up to now, RNA-based fluorogenic modules involving quenched fluorogen have been less characterized. However, even though these modules display a lower affinity than the aforementioned ones (Table 1), they have the great advantage of using very bright and photostable organic dyes as fluorogenic moiety (i.e., symmetrical cynanines [35,77], Sulforhodamines [55] or Rhodamine Green [32]), making them attractive for future developments.

## 3. Applications of RNA-Based Fluorogenic Modules

**Figure 4:**
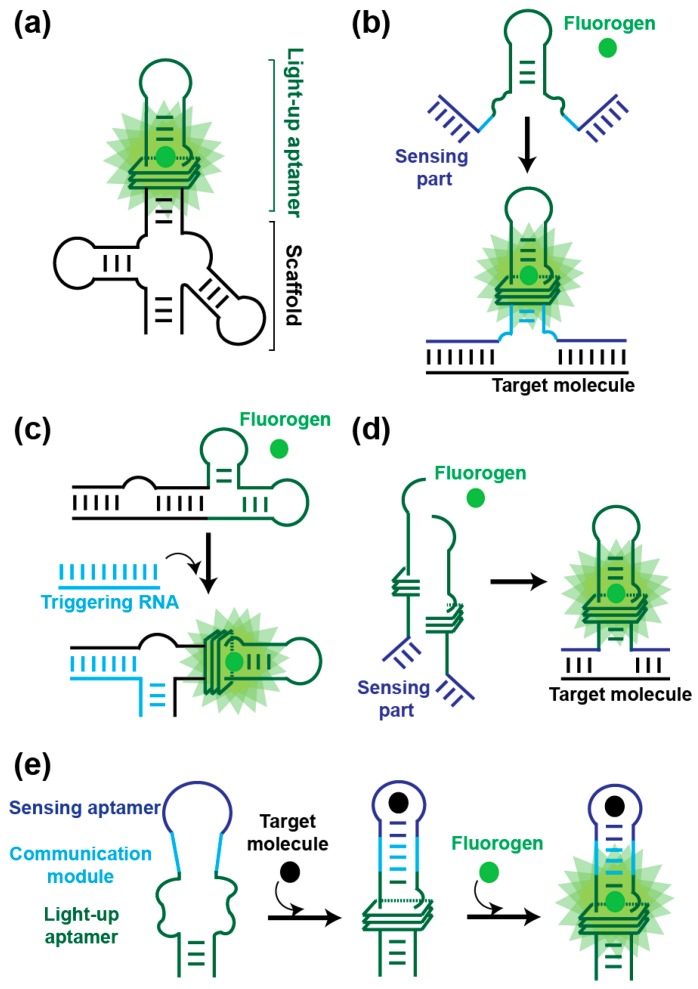
Light-up aptamers engineering and uses thereof. (**a**) Light-up RNA aptamers can be embedded into a scaffold RNA (here a tRNA) to improve folding efficiency and resistance to RNases [29,61]. The construct can either be directly expressed from an independent promoter or inserted into the sequence of a target mRNA. (**b**) Light-up aptamer sensing a target RNA in trans [77,78]. A key helix (light blue) of the light-up aptamer is engineered to weaken the structure of the RNA and destabilized the fluorogen-binding site. Furthermore, sequences complementary to the target nucleic acid (acting as a sensing part) are appended to both ends of the molecule (dark blue). Upon binding to the target molecule (in black), the structure of the light-up moiety is stabilized and its fluorogen-binding capacity is restored, leading to fluorescence emission (green shadow). (**c**) Light-up aptamer sensing a target RNA in trans via strand displacement [79,80,81]. Sequences are appended to both ends of the light-up aptamer and induce an alternative folding preventing the interaction of the aptamer with the fluorogen. However, the binding of the target RNA to the construct induces a conformational change restoring the structure of the light-up aptamer moiety and its fluorogen-binding capacity, leading to fluorescence emission (green shadow). (**d**) Sensing target nucleic acids using split light-up aptamers [82,83]. The aptamer is split into two halves, each possessing a sequence complementary to the target nucleic acid (acting as a sensing part, in dark blue). Whereas both halves cannot form a functional aptamer in the absence of the target nucleic acid, the presence of the latter drives the productive association of both molecules into a functional light-up aptamer able to emit fluorescence. (**e**) Allosteric light-up RNA aptamer [84,85]. The light-up aptamer is engineered to weaken the structure of the RNA and destabilize the fluorogen-binding site. Moreover, the sequence of a sensing aptamer (in dark blue) is inserted into that of the light-up aptamer via a communication module (in light blue). Upon the interaction of the sensing aptamer with a target molecule (e.g., a metabolite or a protein), the structure of the light-up aptamer is stabilized and its fluorogen-binding capacity is restored. Note that all the examples are shown with the Spinach aptamer, but these concepts can be extended to any other light-up aptamer.

The development of RNA-based fluorogenic modules was initially motivated by the need for genetically encoded tools to monitor gene expression and regulation at the RNA level. However, the great flexibility and the ease to engineer these RNA molecules rapidly led to the development of new sets of fluorogenic reporters with applications both in vivo and in vitro (Figure 4).

### 3.1. Live-Cell Imaging of Biomolecules

#### 3.1.1. Live-Cell RNA Imaging

Direct expression of light-up RNA aptamers in cells can be challenging due to their degradation by cell nucleases. Therefore, the molecules are usually expressed inserted into a second RNA such as a transfer RNA (tRNA) [29,86] or a three-way junction [87] acting as a scaffold to both protect the aptamer from rapid degradation and assist its folding (Figure 4a). Expressing such constructs from a strong promoter (e.g., T7 RNA polymerase promoter in *E. coli*, 5S promoter in mammalian cells) allows the expression of abundant non-coding RNAs to be monitored in prokaryotes [29,32,55] and eukaryotes [29,31,34]. Moreover, recent publications reported on the possibility to insert Spinach-derived aptamers into the variable region of bacterial tRNAs [88], within an apical loop of the bacterial 16S ribosomal RNA [89] or in metazoan tRNA introns [90] while preserving the natural functionality of the carrier molecules. Being able to image these non-coding RNAs not only informs on their own expression but it can also be used to diagnose the whole associated pathway (e.g., Pol III transcription, translation, etc.). Spinach was also found suited for imaging messenger RNAs in organisms as diverse as bacteria [91,92,93], yeast [94], mammalian cells [61], viruses [95,96,97] and algae [98]. The limited brightness and poor photostability of Spinach and its derivatives initially limited its use to the imaging of mRNAs either aggregated [61] or labeled with tandem repeats of up to 64 copies of the aptamer [93]. However, an improved understanding the photophysics of DFHBI inactivation allowed devising pulsed excitation-based imaging procedures making it possible to monitor Spinach-labeled RNAs over a much longer period of time [75] and enabling imaging much less abundant mRNAs [93]. Furthermore, using spinning disk confocal microscopy and post-acquisition image analysis algorithms it was possible to track the nuclear export of yeast mRNA labeled with a single copy of Spinach [94]. Interestingly, these studies also revealed that inserting the light-up aptamer into the 3′ untranslated region of the mRNA did not affect the fate (i.e., localization, translation or stability) of the labeled molecule.

Direct insertion of light-up aptamers into target RNAs is mainly limited by the laborious genetics required for modifying the locus of each target gene. This limitation can nevertheless be overcome by using in trans-acting fluorogenic modules that are destabilized forms of the light-up aptamer [77,78] (Figure 4b). In this approach, the aptamer is engineered at two levels. First, a helix of the aptamer is truncated to weaken the structure of the RNA and abolish its capacity to interact with the fluorogen. Second, sequences complementary to the target RNA are appended at both extremities of the helix. Upon binding to the target RNA, the structure of the aptamer is stabilized and its capacity to bind and activate the fluorogen is restored. Provided an unstructured region is accessible in the target RNA, this approach can be used to image both mRNA [77,78] and small non-coding RNA [99]. Moreover, embedding the aptamer into a scaffold RNA and triggering its activation by a strand-displacement mechanism respectively improve the stability of the fluorogenic module and increase signal amplitude [80,81] (Figure 4c). Further improvement in detection specificity and signal amplitude can be obtained by using split aptamers [82,83] (Figure 4d). Recently, a split version of the Broccoli light-up aptamer was also generated and embedded into an AND logic gate for sensing the simultaneous presence of two target RNAs in vivo [100].

#### 3.1.2. Live-Cell Imaging of Metabolites and Proteins

Its great structural flexibility enables RNA to fold into aptamers able to specifically recognize virtually any type of molecule. Moreover, if the interaction between the aptamer and its target leads to a structural remodeling or even the stabilization of RNA structure, then such aptamer can be used for the conception of an allosteric biosensor in which a sensing module is connected to a reporting module via a communication module (Figure 4e). Briefly, the structure of the reporting module (a ribozyme or a light-up aptamer) is destabilized which keeps its function (e.g., RNA cleavage or fluorescence emission) in an off state. However, the presence of the target analyte induces a structural change (or stabilization) of the sensing aptamer that is transmitted to the reporting module via the communication module. The structure of the reporting RNA is in turn stabilized and its function restored. This strategy was originally pioneered using ribozymes as reporting modules [101] and early work with MG aptamer allowed establishing the proof-of-concept experiment using light-up aptamers as reporter [84]. Since then, Spinach RNA-based fluorogenic biosensors have been developed to specifically report on the presence of metabolites such as FMN [84], cyclic-di-GMP [85,102,103,104], cyclic-di-AMP [105], cyclic AMP-GMP [106,107], cyclic-AMP [108], S-adenosylmethionine (SAM) [109], S-adenosyl-l-homocysteine (SAH) [110], Thiamine Pyrophosphate (TPP) [111] and neurotransmitter precursors [112]. The majority of these biosensors are built using riboswitch-derived aptamers. Indeed, since these molecules have naturally evolved to switch RNA structure in cellular environment, these aptamers possess the required structural flexibility and are able to efficiently discriminate their cognate target from closely related analogues contained in the cell. Finally, even though they were less used, allosteric biosensors can also be developed for protein detection [113]. With such biosensor in hands it is then possible to precisely detect and quantify a target molecule in a dynamic way and with single cell resolution. This allows the dynamics of a biological pathway to be monitored, but also to characterize the enzymes involved in this pathway, paving the way for drug discovery applications [105,106,107].

Whereas both sensing and reporting modules have key roles in the proper function of allosteric fluorogenic biosensors, the communication module is probably the most critical element, as it drives the information from one aptamer to the other. Usually, a communication module is rationally designed based on thermodynamic considerations since such module would be extremely challenging to isolate using a SELEX approach. However, the recent advances in high-throughput screening technologies introduced above offer a new way of rapidly identifying optimal communication modules. For instance, a large-scale integration microfluidic device was used to systematically screen a library of 94 different Spinach-based glycine biosensor containing different communication modules while measuring their response to various glycine concentrations [114]. Therefore, a single experiment allows accessing both the fluorescence amplitude of many biosensors as well as their affinity for the glycine and their dynamic range. Consequently, assisting the development of new biosensors by high-throughput screening technologies should both accelerate their development and improve their performances. Finally, one should note that a communication might be dispensable if one simply wants to develop an aptamer-based protein-targeting fluorescent probe. In that case, the binding aptamer can be directly connected to a light-up aptamer as recently exemplified by the use of a fluorogenic cyanine-binding aptamer fused with a VEGF-binding aptamer [27].

### 3.2. In Vitro Applications

The possibility of detecting RNA via fluorogenic assays opened a whole bunch of applications in vitro. First, inserting the sequence of a light-up aptamer into that of another RNA allows monitoring in real-time the in vitro transcription of the construct [35,115] and, doing so, to compute its transcription rate while decorrelating it from the translation rate of an encoded protein. This is of particular importance in experiments aiming at engineering genetic circuits, especially for generating artificial cells [116,117,118]. Being able to monitor transcription activity in an automatable way makes also possible to set-up high-throughput microtiter plate-based screening of drugs targeting RNA polymerase activity [115]. Drug screening actually represents a second important set of applications of light-up aptamers in vitro. For instance, the fluorogenic function of the RNA can be transiently inactivated by modifying (e.g., methylating) a key nucleotide to convert the aptamer into a fluorogenic substrate of a modification-removing enzyme such as demethylases, a class of enzymes involved in several diseases [119]. Monitoring reaction product apparition by the mean of the fluorogenic allosteric biosensors introduced above [110,120,121] represents an alternative way of assaying enzyme activity. In any case, these different strategies can easily be transposed to microtiter plate format to set-up a high-throughput screening workflow [115,119,120] and even an ultrahigh-throughput one by using droplet-based microfluidics [121].

The great ability to predict to some extent the folding of an RNA sequence makes possible the design of molecular circuits composed of two or several RNA (or DNA) elements trapped by folding to keep the circuit silent [122,123]. However, in the presence of one or several target molecules, sensing elements undergo structural rearrangements activating the circuit to compute the information and eventually reports the presence of the targets via engineered light-up aptamer [79,83,124]. Whereas simple circuit made of a split light-up aptamer directly sensing the target molecule would lead to a signal stoichiometric to the input [124], the implementation of an enzyme-free amplification loop [122,123,125] is expected to significantly increase the sensitivity of these assays.

Split and full-length light-up aptamers can also be used to devise in vitro procedures aimed at assisting catalytic RNAs engineering [126,127], tracking RNA-based molecular complexes as well as monitoring the formation of supramolecular assemblies such as those used in RNA nanotechnology [128,129,130,131] directly through fluorescence emission measurements. Moreover, the proper size and integrity of light-up-labeled RNAs can also be verified by gel electrophoresis followed by specific staining of the target RNAs by the fluorogen directly in the gel [87,131]. Finally, light-up aptamers inserted into a target RNA can serve as handles to specifically fish-out the labeled RNA and its associated partners in native conditions by incubating a reaction mixture (e.g., a cell lysate) with a biotinylated fluorogen and purifying the resulting complex on a streptavidin-conjugated resin [132].

## 4. Conclusions

Over the past decade, significant efforts were devoted to the development of new RNA-based fluorogenic modules leading to a toolbox of modules covering the whole visible spectrum (Table 1). Many of these modules are significantly brighter than the widely used GFP and, conversely to their protein counterparts, they do not require oxygen since no maturation step is needed. Therefore, labeling target RNAs with light-up aptamers is not only subjected to a low fluorescent background, but RNAs can also be monitored in conditions in which fluorescent proteins could not be used (e.g., anaerobiosis [104]). Besides direct RNA imaging in vivo, RNA-based fluorogenic modules also found a wide range of applications as briefly reviewed in this article and it is very likely that many new ones shall be developed in a near future (see below).

Interestingly, even though current modules are already pretty efficient and permitted multiple questions to be answered, none of them completely fulfills all the criteria enounced in Section 2 of this review, suggesting that there is still room for improvement in terms of sensitivity and robustness. Indeed, most of the current light-up aptamers were obtained by SELEX using moderate selection pressures and have dissociation constant (so affinity) for the fluorogen of tens of nM or more. Thus far, Mango is the only RNA displaying a very high affinity for its fluorogen (*K_D_* of a few nM), likely resulting from the use of very stringent wash and elution conditions. Therefore, applying the same concept to other systems could yield modules with much higher affinity. This would reduce the amount of dye in the assays and lead to a gain in sensitivity by improving the contrast of the experiment [37]. Moreover, since SELEX-derived RNAs were selected for their capacity to bind the fluorogen rather than their capacity to generate fluorescent complexes, it is likely that the fluorescence of most of the current modules may still be sub-optimal. Indeed, our own experience with Spinach [65] and Mango (on-going work in our lab) RNAs shows that revisiting these aptamers by a screening approach may significantly increase their light-up capacity. Such improvement could result from an increased brightness but also from a higher photostability. Indeed, both properties rely not only on the fluorogen itself but also on capacity of the RNA to properly accommodate it and protect it from unwanted photoisomerization and photobleaching. This last point was recently exemplified by Corn, an RNA isolated by SELEX in tandem with FACS screenings, that forms with DFHO a complex displaying an extraordinary high photostability [34]. This photostability likely results from a caging of the fluorogen preventing its rapid isomerization [73]. This property is clearly contributed by the RNA since lighting-up DFHO with Broccoli did not yield a significant photostability [34]. Therefore, we anticipate that future selection schemes combining conventional SELEX with an (ultra)high-throughput functional screening (e.g., FACS or microfluidic-based screening) together with next-generation sequencing could lead to the efficient discovery of light-up aptamers with superior turn-on and photostabilizing properties. This new generation of molecules should further enlarge the already wide application range of RNA-based fluorogenic modules. For example, they would allow labeling target RNAs with a limited number of aptamer repeats (ideally a single one) to make possible single molecule resolution, thus enabling the tracking of low abundant RNA molecules, for instance using super-resolution microscopy [133]. New generations of fluorogenic modules would not only allow highly sensitive gene expression monitoring, but they could also serve to set-up new drug screening pipelines as well as a variety of analytical platforms such as microarrays developed to sense target molecules with size ranging from ions (recently exemplified by the capacity of Spinach to specifically sense lead [134]) to more complex protein using allosteric biosensors such as those introduce in this review. Finally, whereas this review was focused on RNA aptamers, DNA light-up aptamers were also reported [28,52] and one should consider the possibility of applying the concepts exposed throughout this review to the development of new DNA-based sensors.

## Figures and Tables

**Table 1:** Main RNA-based fluorogenic modules and their properties.

Fluorogen	Light-Up Aptamer	*K_D_* (nM)	Ex./Em. (nm)	ε ^1^ (M^−1^/cm)	Φ^complex 2^	Brightness ^3^	Relative Brightness ^4^	Ref.
GFP	/	/	395/508	21,000	0.770	16.20	0.60	[26]
eGFP	/	/	490/508	39,200	0.680	26.60	1.00	[26]
OTB	DiR2s-Apt	662	380/421	73,000	0.510	37.23	1.40	[27]
Hoescht	Apt II-mini3-4 c	35	345/470	n.a.	0.260	n.a.	n.a.	[28]
DFHBI	Spinach	540	469/501	24,300	0.720	17.50	0.65	[29]
DFHBI-1T	Spinach2	560	482/505	31,000	0.940	29.10	1.10	[30]
DFHBI-1T	Broccoli	360	472/507	29,600	0.940	27.80	1.04	[31]
DFHBI-2T	Spinach2	1300	500/523	29,000	0.120	3.48	0.10	[30]
RG-DN	DNB	4480	507/534	37,350	0.320	11.90	0.44	[32]
TO-1	Mango	3	510/535	77,500	0.140	10.85	0.40	[33]
DFHO	Corn	70	505/545	29,000	0.250	7.25	0.27	[34]
CY3-BHQ1	BHQ apt (A1)	n.a.	520/565	n.a.	n.a.	n.a.	n.a.	[35]
DFHO	Red-Broccoli	206	518/582	35,000	0.340	11.90	0.44	[34]
TMR-DN	DNB	350	555/582	47,150	0.900	42.43	1.60	[32]
SR-DN	DNB	800	572/591	50,250	0.980	49.24	1.80	[32]
DIR	DIR apt	86	600/646	134,000	0.260	34.80	1.30	[36]
Mal. Green	MG aptamer	117	630/650	150,000	0.187	28.00	1.05	[19]
DIR-pro	DIR2s-Apt	252	600/658	164,000	0.330	54.12	2.00	[27]
TO-3	Mango	6–8	637/658	9300	n.a.	n.a.	n.a.	[33]
Patent Blue	SRB apt	23	n.a./665	n.a.	0.034	n.a.	n.a.	[19]

^1^ Absorption coefficient (ε); ^2^ Quantum yield of the complex (Φ^complex^); ^3^ Brightness calculated as Brightness = (ε × Φ^complex^)/1000; ^4^ Brightness expressed relative to eGFP. n.a.: not available. RNA modules were ordered according to their fluorescence emission wavelength.

## Abbreviations

Cy3-BHQ1	Cyanine 3-Black Hole Quencher 1
DIR	Dimethyl Indole Red
DFHBI	3,5-difluoro-4-hydroxybenzylidene imidazolinone
DFHBI-1(2)T	3,5-difluoro-4-hydroxybenzylidene imidazolinone 1 (2) trifluoroethyl
DFHO	3,5-difluoro-4-hydroxybenzylidene imidazolinone-2-oxime
FACS	Fluorescence Activated Cell Sorter
GFP	Green Fluorescent Protein
MG	Malachite Green
OTB	Oxazole Thiazole Blue
RG-DN	Rhodamine Green-DiNitroaniline
SELEX	Systematic Evolution of Ligands by EXponential enrichment
SR-DN	Sulforhodamine-DiNitroaniline
TMR-DN	Tertamethyl rhodamine-DiNitroaniline
TO-1 (3)	Thiazole Orange 1 (3)
